# Suitability of Video Consultations During the COVID-19 Pandemic Lockdown: Cross-sectional Survey Among Norwegian General Practitioners

**DOI:** 10.2196/26433

**Published:** 2021-02-08

**Authors:** Tor Magne Johnsen, Børge Lønnebakke Norberg, Eli Kristiansen, Paolo Zanaboni, Bjarne Austad, Frode Helgetun Krogh, Linn Getz

**Affiliations:** 1 Norwegian Centre for E-health Research Tromsø Norway; 2 Department of Public Health and Nursing Norwegian University of Science and Technology Trondheim Norway; 3 Department of Clinical Medicine Faculty of Health Sciences UiT The Arctic University of Norway Tromsø Norway

**Keywords:** video consultations, digital consultations, eHealth, general practice, primary health care, continuity of care, physician-patient relationship, patient safety, COVID-19, pandemic, telehealth, telemedicine, consultation, safety, cross-sectional, online survey

## Abstract

**Background:**

The COVID-19 pandemic imposed an acute, sharp rise in the use of video consultations (VCs) by general practitioners (GPs) in Norway.

**Objective:**

This study aims to document GPs’ experiences with the large-scale uptake of VCs in the natural experiment context of the pandemic.

**Methods:**

A nationwide, cross-sectional online survey was conducted among Norwegian GPs during the pandemic lockdown (April 14-May 3, 2020). Each respondent was asked to evaluate up to 10 VCs. Basic demographic characteristics of the GPs and their practices were collected. The associations between GPs’ perceived suitability of the VCs, the nature of the patients’ main problems, prior knowledge of the patients (relational continuity), and follow-up of previously presented problems (episodic continuity) were explored using descriptive statistics, diagrams, and chi-square tests.

**Results:**

In total, 1237 GPs (26% of the target group) responded to the survey. Among these, 1000 GPs offered VCs, and 855 GPs evaluated a total of 3484 VCs. Most GPs who offered VCs (1000/1237; 81%) had no experience with VCs before the pandemic. Overall, 51% (1766/3476) of the evaluated VCs were considered to have similar or even better suitability to assess the main reason for contact, compared to face-to-face consultations. In the presence of relational continuity, VCs were considered equal to or better than face-to-face consultations in 57% (1011/1785) of cases, as opposed to 32% (87/274) when the patient was unknown. The suitability rate for follow-up consultations (episodic continuity) was 61% (1165/1919), compared to 35% (544/1556) for new patient problems. Suitability varied considerably across clinical contact reasons. VCs were found most suitable for anxiety and life stress, depression, and administrative purposes, as well as for longstanding or complex problems that normally require multiple follow-up consultations. The GPs estimate that they will conduct about 20% of their consultations by video in a future, nonpandemic setting.

**Conclusions:**

Our study of VCs performed in general practice during the pandemic lockdown indicates a clear future role for VCs in nonpandemic settings. The strong and consistent association between continuity of care and GPs’ perceptions of the suitability of VCs is a new and important finding with considerable relevance for future primary health care planning.

## Introduction

### Background

The digitalization of medical consultations in general practice (family medicine) has in many countries received increasing interest in recent years, with a particular focus on video consultations (VCs) [1-3]. However, the implementation of VCs by general practitioners (GPs) has been relatively slow and supported by limited and inconclusive evidence [4]. The emergence of the COVID-19 pandemic introduced an abrupt and strong stimulus for rapid adoption of VCs in many contexts, producing an effect that was arguably most striking in settings where a digitalization process had already started [5,6].

Prepandemic research on VCs in general practice has mostly been characterized by small studies on selected patient groups [7,8]. Results indicate that VCs might be useful for selected patients or health problems and have the potential for increased patient empowerment, practical convenience, and efficiency gains [9,10]. However, concerns have been raised regarding the clinical quality and suitability of VCs, and both patients and clinicians still consider face-to-face consultations the gold standard [7,8,11,12]. More knowledge is needed about the optimal use of VCs in general practice, from both an organizational and a clinical perspective [13].

In the context of face-to-face consultations, continuity of care [14] has been associated with positive health outcomes for patients, including increased life expectancy [15,16]. There is little knowledge regarding the impact of VCs on the doctor-patient relationship and to what extent a pre-existing doctor-patient relationship might impact the quality and outcome of VCs. The introduction of VCs in situations where GPs deliver continuity of care to their patients (relational continuity) and are familiar with their ongoing health problems (episodic continuity) may have a positive impact compared to situations where continuity of care is not established [17].

### Use of VCs in Norway Before and During the COVID-19 Pandemic

Before the COVID-19 pandemic, around 3% of all GP consultations in Norway were performed digitally [18]. The societal lockdown in Europe in spring 2020 had a strong impact on general practice in Norway. On March 16, 2020, the Norwegian Ministry of Health and Care Services encouraged all GPs to adopt a solution for VCs [19]. The reimbursement system for GPs was also temporarily modified to strengthen the use of medical consultations via text, video, or telephone (Textbox 1). Data from the main providers of VC solutions showed that, while almost all 4822 GPs in Norway installed a solution for VCs, less than 2000 GPs used VCs during the period April 15-May 3, 2020.

During the first phase of the lockdown in mid-March 2020, many GP offices restricted physical access and triaged all contacts via telephone or online communication. Almost 60% (171,169/299,148) of all GP consultations in Norway were performed digitally from March 16 to March 22 (Multimedia Appendix 1). In the last week of March, 25% (34,814/141,501) of all digital consultations were VCs (Multimedia Appendix 2). From May 11 to May 17, the proportion of digital consultations decreased to 30% (85,026/286,419), of which 19% (16,278/85,026) were VCs.

Most GP offices still offered face-to-face consultations for urgent issues. However, digital consultations were conducted even in situations where a physical examination would have been deemed necessary before the COVID-19 lockdown, such as acute abdominal pain or chest pain. At the time of data collection, the main technical solution for VCs in Norway was an external video application not integrated with the GP’s electronic patient record systems. However, the patients’ medical record was available to the GPs during the VCs.

**The Norwegian General Practitioner Scheme.**

The Norwegian health care system is based on the principles of universal access, decentralization, and continuity of care [20]. Since 2001, all Norwegian citizens may sign up with (and change, if desired) a GP, and 99% have chosen to do so. The system is financed by taxation, together with income-related employee and employer contributions and out-of-pocket payments (copayments). Private medical insurance is limited. Although national health care policy is controlled centrally, responsibility for the provision of primary health care is decentralized. GPs act as coordinators of municipal services and gatekeepers to specialized care. On average, a GP has about 1100 patients and often provides other medical services in the municipality one day per week.

### Evaluation of VCs During the COVID-19 Pandemic

In association with the COVID-19 pandemic, recommendations regarding the use of VCs by GPs and their patients have been issued based on clinical expertise and relevant evidence [13,21]. The lockdown led to a rapid uptake of VCs during a very short time period, creating a natural experiment where the effectiveness and suitability of VCs could be explored across a wide range of health problems [5].

Ideally, the large-scale implementation of VCs should have been rigorously monitored by detailed research on both GPs and patients. Due to the pressing circumstances of the lockdown, such systematic evaluation was not deemed feasible. However, conducting a large-scale survey of GPs’ experiences with the use of VCs during the lockdown was achievable and can contribute to filling important knowledge gaps.

The overall aim of this survey was to explore how GPs in Norway perceived the suitability of VCs compared to ordinary face-to-face consultations during the COVID-19 lockdown. In addition to addressing the suitability of VCs across a wide range of health problems (reasons for contact), we were also interested in knowing whether continuity of care (ie, prior knowledge of the patient/problem) had an impact on GPs’ perceptions of VC suitability.

## Methods

### Study Design and Setting

A prospective nationwide online cross-sectional survey was conducted among GPs in Norway during the COVID-19 pandemic lockdown (April 14-May 3, 2020). The survey was addressed to all GPs registered in Norway. Basic demographic characteristics of the GPs and their practices were collected. Each GP also indicated the number of consultations and other activities conducted during the day when the survey was taken.

A central part of the survey addressed GPs’ experiences with VCs before and during the pandemic. Each GP was asked to evaluate up to 10 consecutive (or otherwise unselected) VCs during the COVID-19 lockdown, preferably conducted during the same day. The evaluation of each VC included 13 questions that covered the GPs’ prior knowledge of the patient (relational continuity), whether the reason for contact was a new problem or a follow-up (episodic continuity), the total number of presented problems, the nature of the main problem (as perceived by the GP), the perceived suitability of VC compared to an envisaged face-to-face consultation for the main problem, and actions (one or more) taken by the GP during/after the VC. The GP’s perception of the patient’s satisfaction with the VC, the technical quality of each VC, and their willingness to use VC in a similar situation after the COVID-19 pandemic were also recorded. Finally, the GPs were asked to estimate the overall proportion of VCs they personally envisaged in their practice in a “normalized” future, in light of their accumulated experience with the medium.

Questions were multiple choice with 2-11 alternative answers, depending on the topic. Questions concerning users’ experiences were scored on a 3-point or 4-point Likert scale. Regarding patients’ reasons for contact, a total of 78 alternatives were offered. This list was informed by the International Classification of Primary Care, second edition (ICPC-2), but was less detailed. Regarding actions taken during or after each VC, 16 alternatives were offered. We consulted the Checklist for Reporting Results of Internet E-Surveys (CHERRIES) to develop the survey and report its results [22]. The survey was pilot tested by a panel of experienced GPs. The survey was conducted in Norwegian through the Netigate application. The results have been translated into English for the purpose of publication.

### Data Collection

To obtain access to GPs’ updated contact emails on very short notice, the research team collaborated with Norwegian Health Informatics, a web-based portal that hosts an online clinical decision support product (NEL), to which approximately 98% of all Norwegian GPs subscribe [23]. An invitation was sent to all subscribers by a unique link that ensured both the authenticity and anonymity of the respondents. GPs who did not receive a personal invitation by email or were nonsubscribers of NEL were encouraged by a well-established social media group for GPs to register their email addresses on the Norwegian Health Informatics website. It took approximately 30-60 minutes to complete the survey. Data collection was undertaken in the period April 14-May 3, 2020. Several reminders were sent by email and social media.

### Data Analysis

Results were summarized by descriptive statistics, diagrams, and chi-square tests with 95% CIs. Background data from this survey were compared to available information on all GPs in Norway. The associations between suitability of VCs and relational continuity, episodic continuity, and the nature of the patients’ main problems were explored by diagrams, tables, and chi-square tests. The defined significance level was .05.

Before further analysis was performed, the 78 alternative reasons for contact were merged into 27 more overarching categories (eg, knee, hip, shoulder, and back problems were merged into “musculoskeletal issues”). We also merged “better” and “same” into one category regarding suitability. Data were analyzed in IBM Statistical Package for the Social Sciences (SPSS; Version 26.0, IBM Corp).

### Ethics

Ethical considerations were included in all phases of the survey. Participating GPs were informed that participation was voluntary and anonymous. We did not elicit sensitive information or demographic characteristics that could reveal the identity of the GPs. For the evaluated VCs, we did not elicit patients’ age, sex, specific diagnoses, or other sensitive or person-related information. Distribution of the survey to GPs’ email addresses was handled by an independent party (Norwegian Health Informatics). No linkage key was established, and participants’ IP numbers were not accessible to any party. Further approvals were thereby not required, according to Norwegian health research legislation, verified by the Norwegian Centre for Research Data (NSD).

## Results

### Characteristics of the GPs

A total of 1237 GPs participated in the survey, representing 26% (1237/4822) of the total GP population in Norway [24]. Of these, 1000 (81%) answered that they were equipped to offer VCs at the time of the survey, and 855 contributed evaluations of at least one VC (Table 1).

On average, each GP conducted 20 consultations during the surveyed working day. Of these, 6.9 (34.5%) were face-to-face consultations, 5.3 (26.5%) were VCs, 3.3 (16.5%) were text-based e-consultations, and 4.5 (22.5%) were telephone consultations. Of the 855 participants, 74 (9%) had no face-to-face consultations on the study day. Most of the respondents (80%; 687/855) did not have any experience with the use of VCs before the COVID-19 pandemic.

**Table 1:** Characteristics of the 855 general practitioners who evaluated one or more video consultations.

Characteristics of the general practitioners (N=855)		Participants, n (%)
**Gender**		
	Female	480 (56.2)
	Male	368 (43.0)
	No answer	7 (0.8)
**Experience in years as a general practitioner**		
	0-5	174 (20.3)
	6-10	189 (22.1)
	11-20	287 (33.6)
	>20	205 (24.0)
**Inhabitants of the municipality of practice**		
	<10,000	130 (15.2)
	10,000-50,000	295 (34.5)
	50,000-100,000	143 (16.7)
	100,000-500,000	217 (25.4)
	>500,000	70 (8.2)
**Experience with video consultations before the COVID-19 pandemic**		
	None	687 (80.3)
	Limited (1-50 video consultations)	123 (14.4)
	Relatively good (>50 video consultations)	45 (5.3)

### Description of the VCs

On average, each GP provided an evaluation of 3.8 VCs. The final data set included 3484 unique VCs between GPs and patients (Table 2).

In most VCs (79%; 2760/3484), the GP knew the patient well beforehand, while the GPs described only 8% (276/3484) of the patients as previously unknown. More than half of the consultations (55%; 1921/3481) were a follow-up of a previous problem. On average, 1.9 (median 2.0) problems/issues were discussed during each consultation. Half of the VCs (51%; 1766/3476) were considered to have similar or even better suitability to assess the main reason for contact compared to face-to-face consultations. For 15% (514/3476) of the VCs, the GPs expressed concern that they might not have detected potential signs of serious illness. The lack of opportunity to physically examine the patient was reported as a “major loss” or “some loss” in 25% (884/3475) and 36% (1232/3475) of the VCs, respectively. In 85% (2967/3475) of cases, the GPs perceived that the patient was satisfied with the VC. The technical quality was considered good in 90% (3118/3475) of the VCs. Half of the GPs (1704/3475) considered it realistic to handle a similar issue by VC after the COVID-19 pandemic.

**Table 2:** Characteristics of the 3484 recorded video consultations. Missing answers (ranging from 0-9 general practitioners per question) are not displayed.

Characteristics of the video consultations (N=3484)		Values, n (%)
**General practitioner’s pre-existing knowledge of the patient**		
	Very good	1788 (51.3)
	Good	972 (27.9)
	Some	448 (12.9)
	None	276 (7.9)
**Main reason for contact**		
	New problem	1560 (44.8)
	Follow-up	1921 (55.1)
**Total number of contact reasons discussed**		
	1	1471 (42.2)
	2-4	1930 (55.4)
	>4	75 (2.2)
**Suitability of** **video consultation** **compared to a face-to-face consultation for the same reason**		
	Better or same	1766 (50.7)
	Worse	1709 (49.1)
**Suitability of** **video consultation** **to assess the severity of the main reason for contact compared to a face-to-face consultation**		
	Better or same	1767 (50.7)
	Worse	1709 (49.1)
**Loss from not being able to examine the patient physically**		
	No loss	1359 (39.0)
	Some loss	1232 (35.4)
	Major loss	884 (25.4)
**Concern about not picking up signs of serious illness**		
	Not worried	2009 (57.7)
	Neutral	953 (27.3)
	Worried	514 (15.0)
**General practitioner’s perception of patient satisfaction with** **video consultation**		
	Very satisfactory	988 (28.4)
	Satisfactory	1979 (56.8)
	Unsatisfactory	368 (10.6)
	Do not know	140 (4.0)
**General practitioner’s satisfaction with technology (connection, sound, image)**		
	Very satisfactory	1433 (41.1)
	Satisfactory	1685 (48.4)
	Unsatisfactory	273 (7.8)
	Video consultation terminated due to technical problems	84 (2.4)
**Motivation to conduct a video consultation for a similar health problem (reason for contact) in a nonpandemic future**		
	Yes	1704 (48.9)
	Do not know	768 (22.0)
	No	1003 (28.8)

### Suitability of VCs and Continuity of Care

**Figure 1:**
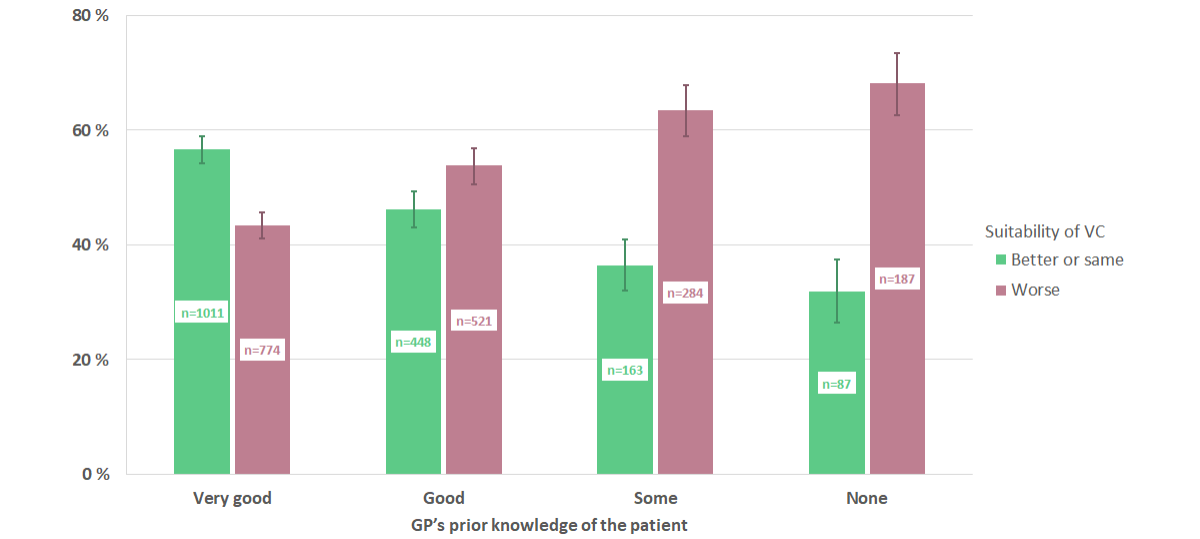
Association between relational continuity (previous knowledge of the patient) and general practitioners' perceived suitability of video consultations compared to an envisaged face-to-face consultation for the same issue (95% CIs are illustrated by lines). GP: general practitioner; VC: video consultation.

The association between GPs’ perceptions of the suitability of VCs and their previous knowledge of their patients (relational continuity) is presented in Figure 1. When the GPs knew their patients “very well” beforehand, VCs were considered as having “better” or “same” suitability, compared to face-to-face consultations in 57% (1011/1785) of cases. When the patient was “unknown,” the corresponding suitability rate dropped to 32% (87/274). The difference in proportions of better/same and worse suitability of VCs between the groups of relational continuity was statistically significant (*χ*^2^_3_=105.3, *P*<.001).

**Figure 2:**
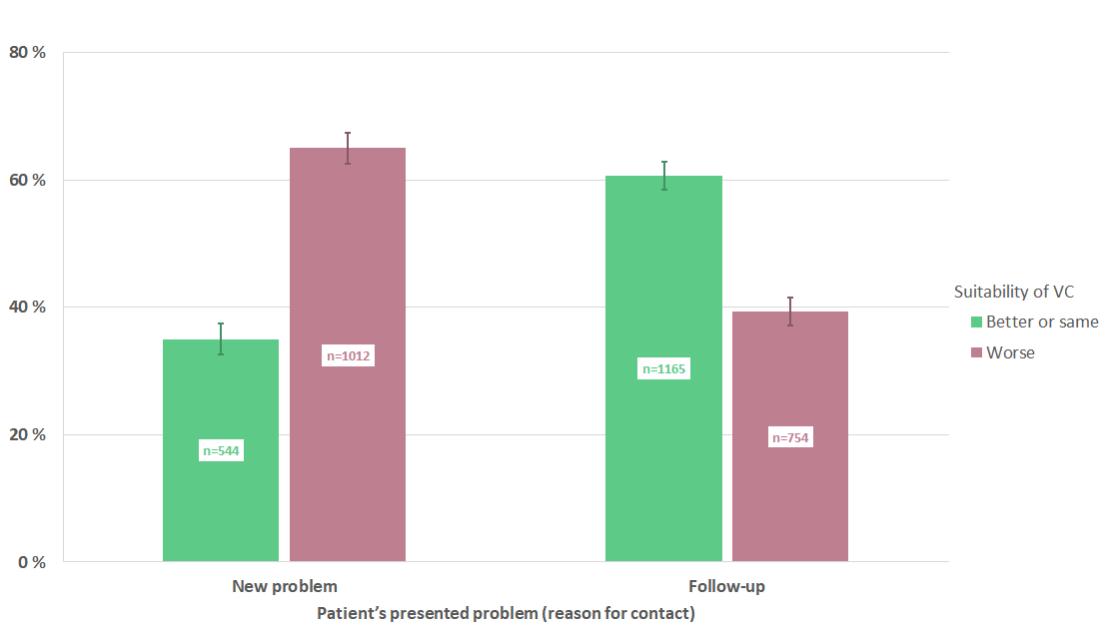
Association between episodic continuity (new problem or follow-up of previously defined problem) and general practitioners' perceived suitability of the video consultations, compared to envisaged face-to-face consultations for the same problem (95% CIs are illustrated by lines). VC: video consultation.

In Figure 2, we present the association between the GPs’ perceptions of suitability of VCs and their previous knowledge of a given patient’s presented problem (episodic continuity). VCs were considered better/same compared to envisaged face-to-face consultations in 61% (1165/1919) of follow-up consultations, as opposed to 35% (544/1556) when the patient presented a new problem. The difference in proportions of better/same and worse suitability of VCs between the groups of episodic continuity was statistically significant (*χ*^2^_1_=227.9, *P*<.001).

In Table 3**,** we have combined relational and episodic continuity of care. We present the proportion of VCs where the suitability is considered better or the same, compared to envisaged face-to-face consultations for the same contact reason. When the GP’s prior knowledge of a patient was “very good” or “good” and the problem was a “follow-up,” 62% (1070/1719) of the VCs were considered equally or better suited than envisaged face-to-face consultations. The corresponding proportion drops to 30% (155/521) when the GP’s prior knowledge of the patient was “some” or “none” and the problem was “new.” The differences in proportion of the suitability of VCs in Table 3 are statistically significant (*P*<.001).

**Table 3:** Video consultations perceived by the GPs as equally or better suited compared to an envisaged face-to-face consultation, shown as combinations of pre-existing knowledge of the patient (relational continuity) and the main reason for contact (episodic continuity).

Main reason for contact	Pre-existing knowledge of patient			
	Very good/good (n=2754)		Some/none (n=721)	
	Number of video consultations, n/N	Proportion, % (95% CI)	Number of video consultations, n/N	Proportion, % (95% CI)
New problem (n=1556)	389/1035	38 (35-41)	155/521	30 (26-34)
Follow-up (n=1919)	1070/1719	62 (60-65)	95/200	48 (41-54)

### Suitability of VCs and Reasons for Contact

**Figure 3:**
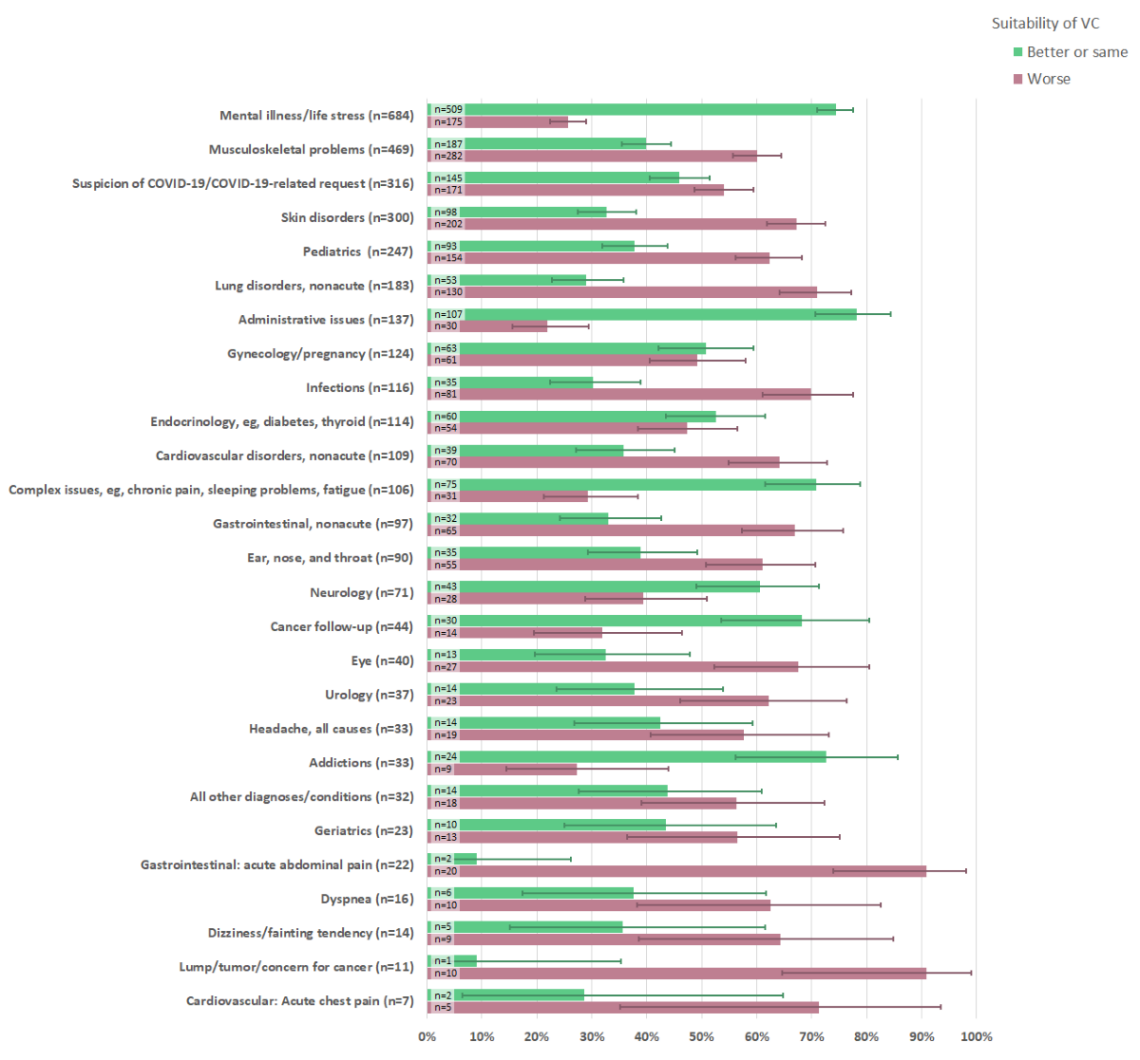
General practitioners' perceived suitability of video consultations compared to an envisaged face-to-face consultation for the same issue, according to the nature of the main problem/reason for contact. The contact reasons are presented in decreasing frequency (n) from top to bottom, and 95% CIs are illustrated by lines. VC: video consultation.

GPs considered VCs to be equally or better suited for several reasons for contact, compared to envisaged face-to-face consultations (Figure 3); examples include mental illness/life stress (509/684; 74%, 95% CI 71%-78%) and various administrative purposes (107/137; 78%, 95% CI 70%-84%). On the other hand, other issues, including musculoskeletal problems (187/469; 40%, 95% CI 36%-44%) and skin disorders (98/300; 33%, 95% CI 28%-38%), were regarded as less suitable for VCs. VCs were also considered less suitable in situations involving acute chest pain, stomach pain, and fear/investigation of a potential new cancer.

Furthermore, for each main reason for contact, the perceived suitability of VCs differed according to episodic continuity of care (Table 4). For instance, the suitability of VCs for skin disorders was 30% (69/234) for a new problem and 44% (29/66) for a previously discussed problem. Corresponding numbers were 18% (33/183) and 54% (154/286) for musculoskeletal problems, and 20% (4/20) and 77% (39/51) for neurology disorders. The impact of relational continuity of care on the suitability of VCs for individual health problems is not reported due to low statistical power (few unknown patients).

**Table 4:** Suitability of video consultations compared to face-to-face consultations, association with main reason for contact (new problem/follow up), and the 20 most common issues/presented problems, N=3350.

Issue/presented problem	Main reason for contact							
	New problem (n=1484)				Follow-up (n=1866)			
	Better or same (n=529)		Worse (n=955)		Better or same (n=1140)		Worse (n=726)	
	Contacts, n/N	Proportion, % (95% CI)	Contacts, n/N	Proportion, % (95% CI)	Contacts, n/N	Proportion, % (95% CI)	Contacts, n/N	Proportion, % (95% CI)
Mental illness/life stress	67/105	64 (54-73)^a^	38/105	36 (28-46)^a^	442/579	76 (73-80)^a^	137/579	24 (20-27)^a^
Musculoskeletal problems	33/183	18 (13-24)^a^	150/183	82 (76-87)^a^	154/286	54 (48-60)	132/286	46 (40-52)
Suspicion of COVID-19 or COVID-19–related	99/222	45 (38-51)	123/222	55 (49-62)	46/94	49 (39-59)	48/94	51 (41-61)
Skin disorders	69/234	30 (24-36)^a^	165/234	71 (64-76)^a^	29/66	44 (32-56)	37/66	56 (44-68)
Children	52/173	30 (24-37)^a^	121/173	70 (63-76)^a^	41/74	55 (44-66)	33/74	45 (34-56)
Lung	27/96	28 (20-38)^a^	69/96	72 (62-80)^a^	26/87	30 (21-40)^a^	61/87	70 (60-79)^a^
Administrative issues	45/57	79 (67-88)^a^	12/57	21 (12-33)^a^	62/80	78 (68-86)^a^	18/80	23 (14-33)^a^
Gynecology and pregnancy	24/50	48 (35-62)	26/50	52 (38-65)	39/74	53 (41-64)	35/74	47 (36-59)
Infection	25/84	30 (21-40)^a^	59/84	70 (60-79)^a^	10/32	31 (17-48)	22/32	69 (52-83)
Endocrinology	6/15	40 (19-65)	9/15	60 (35-81)	54/99	55 (45-64)	45/99	46 (36-55)
Cardiovascular	2/37	19 (9-34)^a^	30/37	81 (66-91)^a^	32/72	44 (33-56)	40/72	56 (44-67)
Complex issues and disorders	10/20	50 (29-71)	10/20	50 (29-71)	65/86	76 (66-84)^a^	21/86	24 (16-34)^a^
Gastrointestinal	12/47	26 (15-39)^a^	35/47	75 (61-85)^a^	20/50	40 (27-54)	30/50	60 (46-73)
ENT (ear, nose, and throat)	23/68	34 (23-46)^a^	45/68	66 (54-77)^a^	12/22	55 (34-74)	10/22	46 (26-66)
Neurology	4/20	20 (7-41)^a^	16/20	80 (59-93)^a^	39/51	77 (64-86)^a^	12/51	24 (14-36)^a^
Cancer follow-up	2/3	67 (18-96)	1/3	33 (4-82)	28/41	68 (53-81)^a^	13/41	32 (19-47)^a^
Eye	12/36	33 (20-50)^a^	24/36	67 (51-80)^a^	1/4	25 (3-72)	3/4	75 (28-97)
Urology	9/22	41 (23-62)	13/22	59 (39-78)	5/15	33 (14-58)	10/15	67 (42-86)
Headache	1/8	13 (1-45)^a^	7/8	88 (55-99)^a^	13/25	52 (33-71)	12/25	48 (30-67)
Intoxication and addiction	2/4	50 (12-88)^a^	2/4	50 (12-88)^a^	22/29	76 (58-89)^a^	7/29	24 (12-42)^a^

^a^Nonoverlapping CIs.

### Suitability of VCs and Actions Taken in the VCs

A total of 7647 actions were registered by the GP during/after the VCs (Table 5). The most common action (47%; 1646/3476) was “comprehensive advice and guidance,” followed by planning a new VC or text-based e-consultation (34%; 1197/3476).

**Figure 4:**
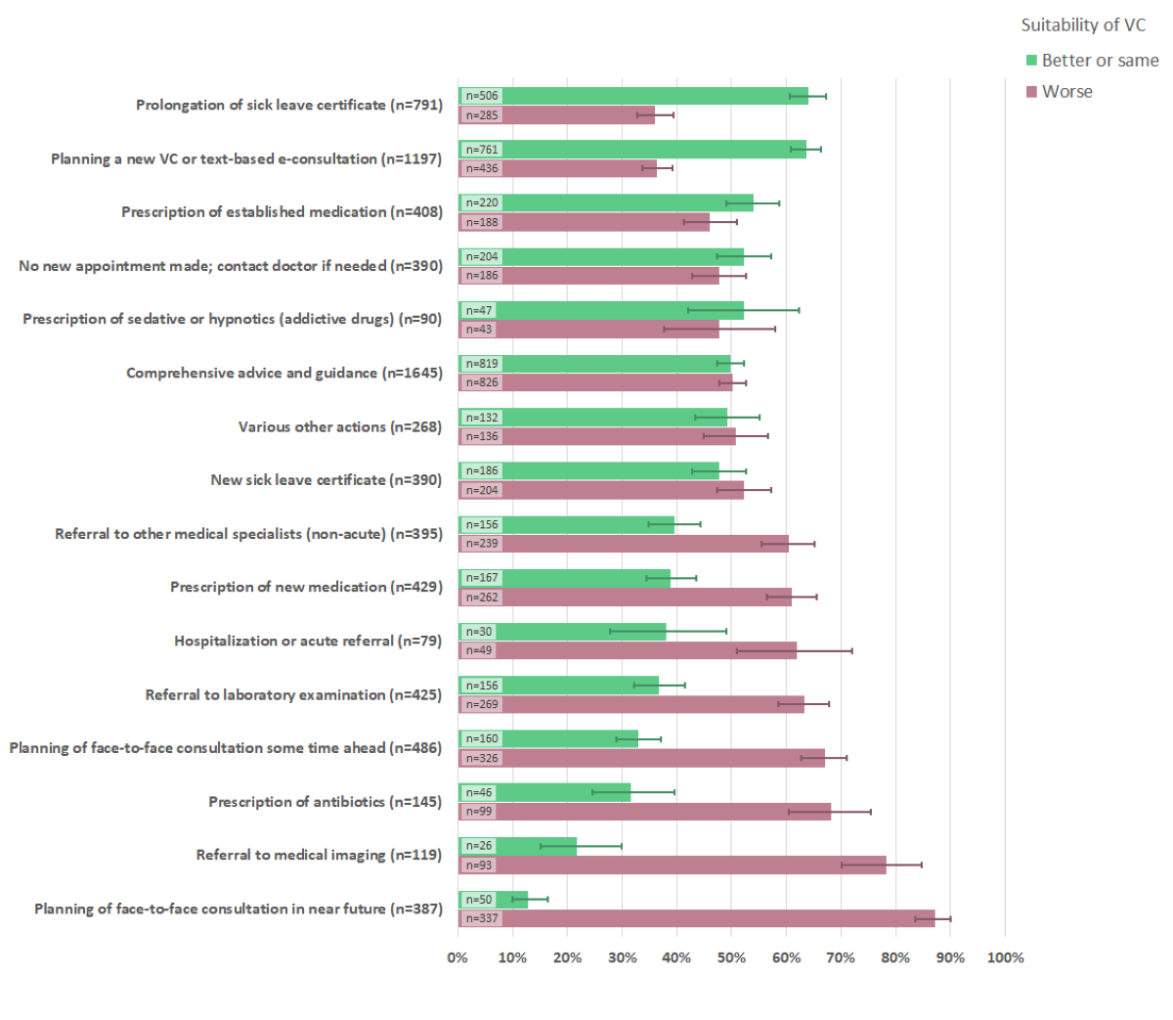
Associations between general practitioners' perceived suitability of video consultations and actions taken during/after the consultation. The actions taken are sorted by general practitioners' perceived suitability (better or same) of the video consultation in question, displayed in decreasing order. 95% CIs are illustrated by lines. VC: video consultation.

VCs were deemed to be less suitable in situations where a physical follow-up was subsequently planned (ie, a face-to-face consultation in the near future, referrals to medical imaging, laboratory examinations, and hospitalizations; Figure 4). Prescription of antibiotics or new medications were also situations where VCs were considered less suitable. Conversely, VCs were perceived as suitable for prolonging sick leaves.

**Table 5:** Action taken during/after the 3484 evaluated video consultations. Several actions could be registered for each video consultation.^a^

Type of actions taken during/after the video consultation (N=7647)	Count, n	%
Comprehensive advice and guidance	1646	47
Planning a new video consultation or text-based e-consultation	1197	34
Prolongation of sick leave certificate	791	23
Planning of face-to-face consultation some time ahead	486	14
Prescription of new medication	429	12
Referral to laboratory testing	425	12
Renewal of established medication	408	12
Referral to other medical specialists (nonacute)	395	11
New sick leave certificate	391	11
No new appointment made; contact doctor if needed	390	11
Planning of face-to-face consultation in near future	388	11
Various other actions	268	8
Prescription of antibiotics	145	4
Referral to medical imaging	119	3
Prescription of sedatives or hypnotics (addictive drugs)	90	3
Hospitalization or acute referral	79	2

^a^In total, 8 evaluated video consultations did not have a registered action.

### Envisaging the Place for VCs in a Normalized Future

Based on their accumulated experience with VCs, participating GPs estimated that on average, 19% of all their consultations (median 20%) could be conducted via video in a normalized, nonpandemic future. Further analyses showed that this estimate was not associated with the GP’s geographic context, previous experience with VCs, age, or gender.

## Discussion

### Principal Findings

This online, cross-sectional survey conducted among GPs in Norway during the COVID-19 pandemic lockdown provides new knowledge about GPs’ experiences with large-scale uptake of VCs within the context of an established national primary health care system. Data collection took place in a phase of the lockdown when infection rates had peaked and most GPs had adapted their working practices, including implementation of VCs, to the pandemic circumstances.

Overall, VCs were perceived as equally or even more suitable than face-to-face consultations in about half of the 3484 evaluated cases. VCs appeared significantly more appropriate in the context of an established doctor-patient relationship and in relation to a previously defined reason for contact. GPs expressed concern about immediate patient safety (ie, the risk of missing signs of serious disease) in 15% (514/3476) of the evaluated cases.

### Validating the Concept of Suitability

Most of the survey was developed around the perceived suitability of VCs in general practice, compared to ordinary face-to-face consultations. It was therefore essential to ensure that the participating GPs had a shared understanding of the concept of suitability. Face validity of the term was assessed when the survey was pilot tested and was found to be satisfactory. Moreover, GPs were instructed to evaluate the suitability of VCs without emphasis on the evident gain of reducing the risk of viral contamination. Furthermore, the analysis of the association between perceived suitability and actions taken in the VCs contributed to the validation of the suitability concept. Consultations categorized with low suitability for VCs often had an “unfinished” character, thus requiring further clinical investigations and/or referrals that could have been more effectively managed in an ordinary face-to-face consultation (Figure 4).

### Impact of Continuity of Care on the Perceived Suitability of VCs

A central finding in this survey was a strong and consistent association between both relational and episodic continuity of care and GPs’ perceptions of the suitability of VCs (Figures 1 and 2, Tables 3 and 4). When GPs knew the patients very well beforehand, VCs were considered equally or better suited than face-to-face consultations in 57% (1011/1785) of cases, as opposed to 32% (87/274) when the patient was previously unknown. Moreover, the suitability rate for follow-up consultations was 61% (1165/1919), while for new problems it was 35% (544/1556)*.* It is important to note that these findings arose within the Norwegian GP list system and that the patient was totally new to the GP in only 8% (276/3484) of the evaluated VCs. To our knowledge, our study presents the first large-scale analysis of the impact of continuity of care on GPs’ perceptions regarding the clinical suitability of VCs. It remains to be seen whether the quantifiable, beneficial outcomes of continuity of care found in physical consultation settings can be replicated in relation to VCs. Beyond this question, our results are in good accordance with previous findings, deliberations, and recommendations regarding quality potentials and pitfalls related to VCs [10,21,25,26].

### Suitability of VCs for Specific Contact Reasons

The predefined list of medical issues used to categorize the main contact reason (presented problem) for each evaluated VC was developed for the purpose of this survey to provide a clinically relevant overview while being general enough to safeguard patient anonymity. We acknowledge that the opportunity to categorize only one (perceived by the GP as the main) reason for contact underrates the clinical complexity of the evaluated VCs, as many of them dealt with two or more health problems (Table 2). In our VC material, mental problems and life stress, musculoskeletal disorders, and COVID-19–related contacts occurred most frequently. When most countries in Europe imposed a lockdown in March 2020, VCs were predicted to be potentially useful for consulting about COVID-19 for people with heightened anxiety, mild symptoms suggestive of COVID-19, or more severe symptoms [13]. The results from our survey confirm these predictions.

Moreover, under the given circumstances, we found that VCs were suitable for a variety of mental health problems, along with other chronic or complex issues such as chronic pain, tiredness, sleeping problems, follow-up of established cancer treatment, and administrative purposes (Figure 3). These results are also in line with previous expectations that VCs could replace in-person visits for contacts related to chronic disease reviews, counselling, or other talking therapies, or administrative appointments such as sick leave certificates [13,27]. Chronic and complex problems are prevalent in general practice and typically involve several consultations with the GP over time [28]. In the presence of continuity of care, such problems can typically be handled in a collaborative dialogical process between doctor and patient. Further research might elucidate to what extent effective clinical relationships can be established and maintained through VCs.

Contacts related to skin problems, pediatric issues, and acute and potentially severe health problems (such as abdominal pain, chest pain, and respiratory difficulties) were found to be the least suitable for VCs. Real-time video might be a feasible alternative to face-to-face and store-and-forward (asynchronous) consultations for selected skin issues [29,30]. However, the evidence regarding the diagnostic accuracy of VCs is still weak [30]. Particular caution must be shown in situations where patient safety might be at risk, including the evaluation of potential malignancy [29-31]. Figure 3 provides an informative overall overview of GPs’ perceived suitability of VCs for various contact reasons, but there is a clear need for more research to refine the knowledge about suitability for VCs in a normalized setting.

Our material included only 23 cases where the main problem was categorized as “geriatric,” and the majority of these cases were classified with low suitability for VC. This aligns well with previous concerns that VCs may not be suitable for use by frail, older patients [8]. Overall, we believe that our results regarding clinical suitability are in good accordance with previous research and recommendations for VCs [13,26], as well as with a recent paper on the interpretative and contextualized nature of diagnostic “knowing” in general practice [32].

### Characteristics of the GPs

Our study participants constitute approximately 26% (1237/4822) of all registered GPs in Norway. Their demographic characteristics are well representative of the GP population in Norway, with the exception of a slightly higher representation of younger doctors [24]. Since the survey was distributed through a unique link to each respondent, multiple responses from the same source can be ruled out.

Before deciding to participate in the survey, invited GPs were informed that VCs would be a central topic, but not a prerequisite for participation. This may nonetheless have attracted GPs with a positive attitude toward digital solutions. As mentioned in the introduction, many GPs who acquired VC equipment in relation to the pandemic lockdown hardly used it in practice. As previously argued, the implementation of VCs is not mainly a simple question of equipment installation, but a complex process of integrating a new consultation modality into established working routines [33]. As explained in the introduction, our estimates suggest that almost 50% of Norwegian GPs who used VCs in April 2020 participated in this survey, which is a clear strength.

### Selection Mechanisms Behind the Evaluated VCs

On average, each participating GP performed five VCs on the day of the survey, while typically evaluating three of these. The survey instructed the GPs not to purposefully select particular types of VCs for evaluation. Although we cannot rule out possible selection bias, it is unlikely that GPs would deliberately restrict their contribution to specific cases. The risk of substantial confirmation bias is therefore considered low.

However, other important selection mechanisms may have influenced our VC material. The study was undertaken in a lockdown period when patients were typically recommended to consult their GPs digitally or by phone before a physical consultation could be considered. In some GP offices, patients could book a VC directly through an online booking system. Beyond this, there were no formal guidelines on how to select patients for VCs. Irrespective of variations in booking systems, many GP offices would arrange physical consultations for selected urgent health problems. Nevertheless, our material includes numerous cases that would normally be considered poorly suited for VC (eg, acute abdomen and chest pain). This aligns well with the fact that participating GPs relatively often reported that they missed the opportunity to perform a physical examination (61% of cases) or expressed concern that they might have misjudged the severity of the presented health problem (15% of cases). Our results cannot be generalized to the use of VCs under nonpandemic circumstances, but we believe they have substantial external validity.

### Current Status and Future Perspectives

The COVID-19 pandemic has led to a crisis for many people and organizations worldwide. At the same time, it has offered opportunities to rethink what is important in general practice and really put new consultation modalities to the test [34]. Overall, our study indicates that VCs have a definite role in future general practice. A crucial question is how to apply VCs to appropriate purposes in an organizationally sustainable manner. On the positive side, VCs can facilitate access and provide rapid solutions in well-selected situations. They can also enhance access to care for disadvantaged or vulnerable patients who are digitally literate but reluctant to visit the GP office. On the flip side, too liberal a use of digital consultations may contribute to a more transactional and less relational way of dealing with deeply human issues, potentially undermining the health-promoting potential of person-centered, longitudinal care [17,25]. Furthermore, it is important to monitor the impact of the implementation of digital consultation modalities on the total clinical workload [35,36]. Patients’ thresholds for contacting the GP for self-limiting conditions may decrease when digital options are easily accessible. Ineffective selection of cases for VCs might trigger physical follow-up consultations for the same problem, leading to increased workloads without associated clinical benefit.

In our study, GPs experienced technical difficulties in 10% of the consultations, somewhat less than a comparable study performed in New York City in March 2020, where technical problems were reported in 13% of consultations [37]. This indicates that more seamless integration of VCs with GPs’ electronic record systems still remains a priority. Despite some technical challenges, the GPs reported that 85% of the patients seemed satisfied with the VCs, in accordance with existing knowledge on patient experience with VCs [9,37,38]. In about half the consultations, the GPs in our survey also deemed it realistic to handle a similar issue by VC in a future, normalized situation. At first glance, this might reflect a strong belief in digitalized health care. However, our findings emerged in an extraordinary setting, where many patients and GPs reportedly felt gratitude for being able to “see” each other at all, despite the ominous threat of contagion and dramatic lockdown measures.

Comparison of clinical practice during lockdown with practice-as-usual reveals numerous discrepancies, both obvious and subtle [39]. Experience-based insight into these differences and their impact on the effectiveness and quality of care is likely to explain why the typical GP in our survey envisaged conducting only about 20% of consultations by video in a normalized future. This prediction is substantially lower than the level of enthusiasm otherwise reflected in our material but would nevertheless represent an important organizational leap for Norwegian general practice as a whole.

### Conclusion

Our study of VCs performed in Norwegian general practice during the pandemic lockdown indicates a future role for VCs in future, nonpandemic settings. The strong and consistent association between continuity of care and GPs’ perceptions of the suitability of VCs is a new and important finding with considerable relevance for future primary health care planning. In accordance with existing literature and guidance, GPs’ perceived suitability of VCs varied considerably across reasons for contact and presented health problems. The findings cannot be directly generalized beyond the specific context of the pandemic lockdown, but nevertheless provide interesting results regarding the performance of VCs for different reasons for contact and clinical conditions. The results indicate that GPs still consider the physical examination a crucial element of many consultations to enhance both diagnostic accuracy and quality of care in a wider sense. Reflecting on the accumulated experience with VCs, most participating GPs envisaged conducting 20% of their consultations by video in a future, nonpandemic setting.

## Appendix

Multimedia Appendix 1 Consultations with general practitioners in Norway during the COVID-19 lockdown. The shaded period corresponds to the period of the data collection. April 6-12, 2020, was the Easter holiday week. Data obtained from Norwegian general practitioners' reimbursement claims and The Norwegian Directorate of eHealth.

Multimedia Appendix 2 Digital consultations with general practitioners in Norway during the COVID-19 lockdown. The shaded period corresponds to the period of data collection. April 6-12, 2020, was the Easter holiday week. Data from Norwegian general practitioners' reimbursement claims and The Norwegian Directorate of eHealth.

## Abbreviations

GP: general practitioner
VC: video consultation
