# Supplementary figures and images for: Suitability of Video Consultations During the COVID-19 Pandemic Lockdown: Cross-sectional Survey Among Norwegian General Practitioners
Source: J Med Internet Res. 2021 Feb 8;23(2):e26433. doi: 10.2196/26433 (PMC7872327; doi:10.2196/26433)

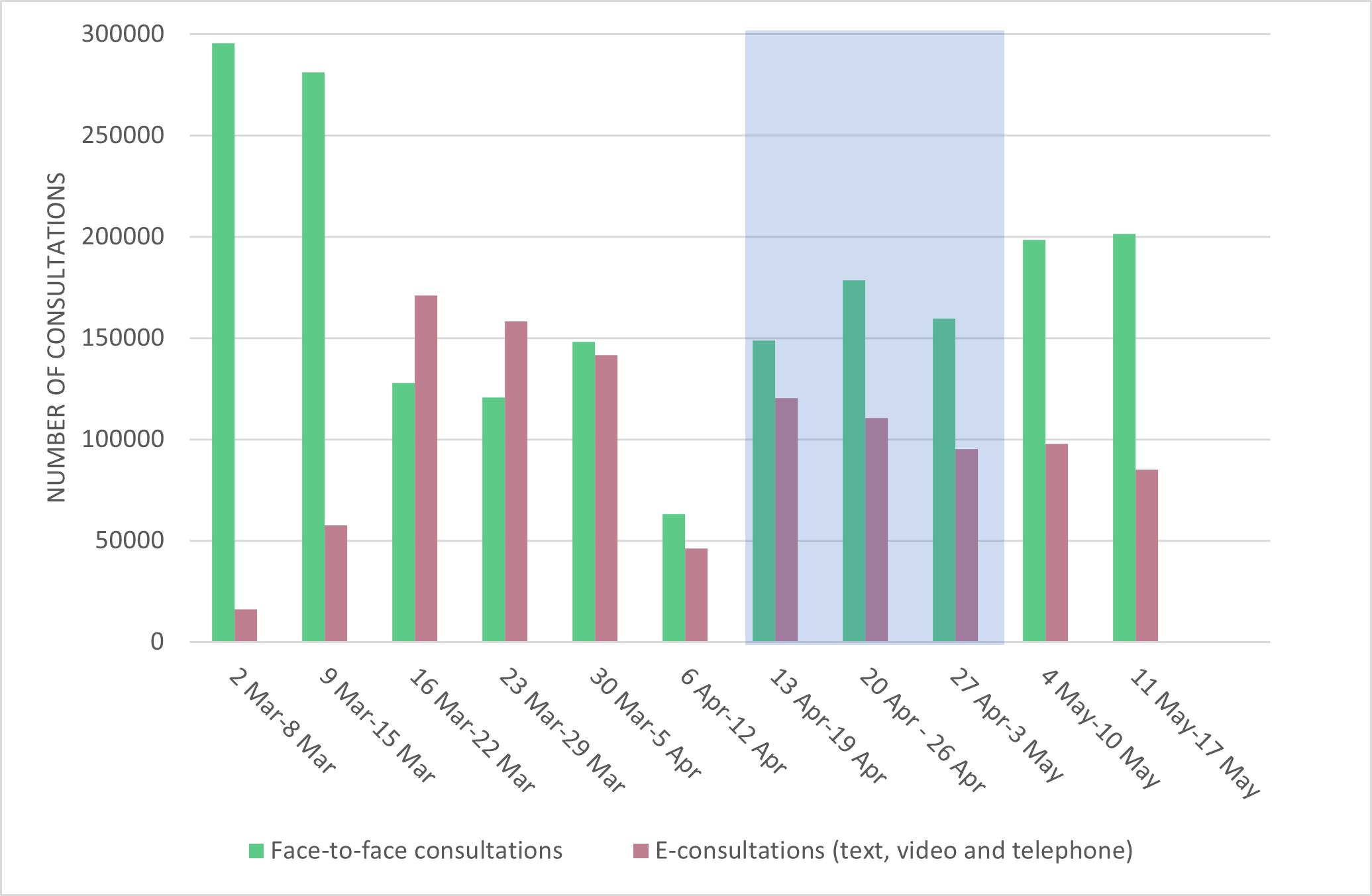

Supplement: Multimedia Appendix 1 [file jmir_v23i2e26433_app1.png]

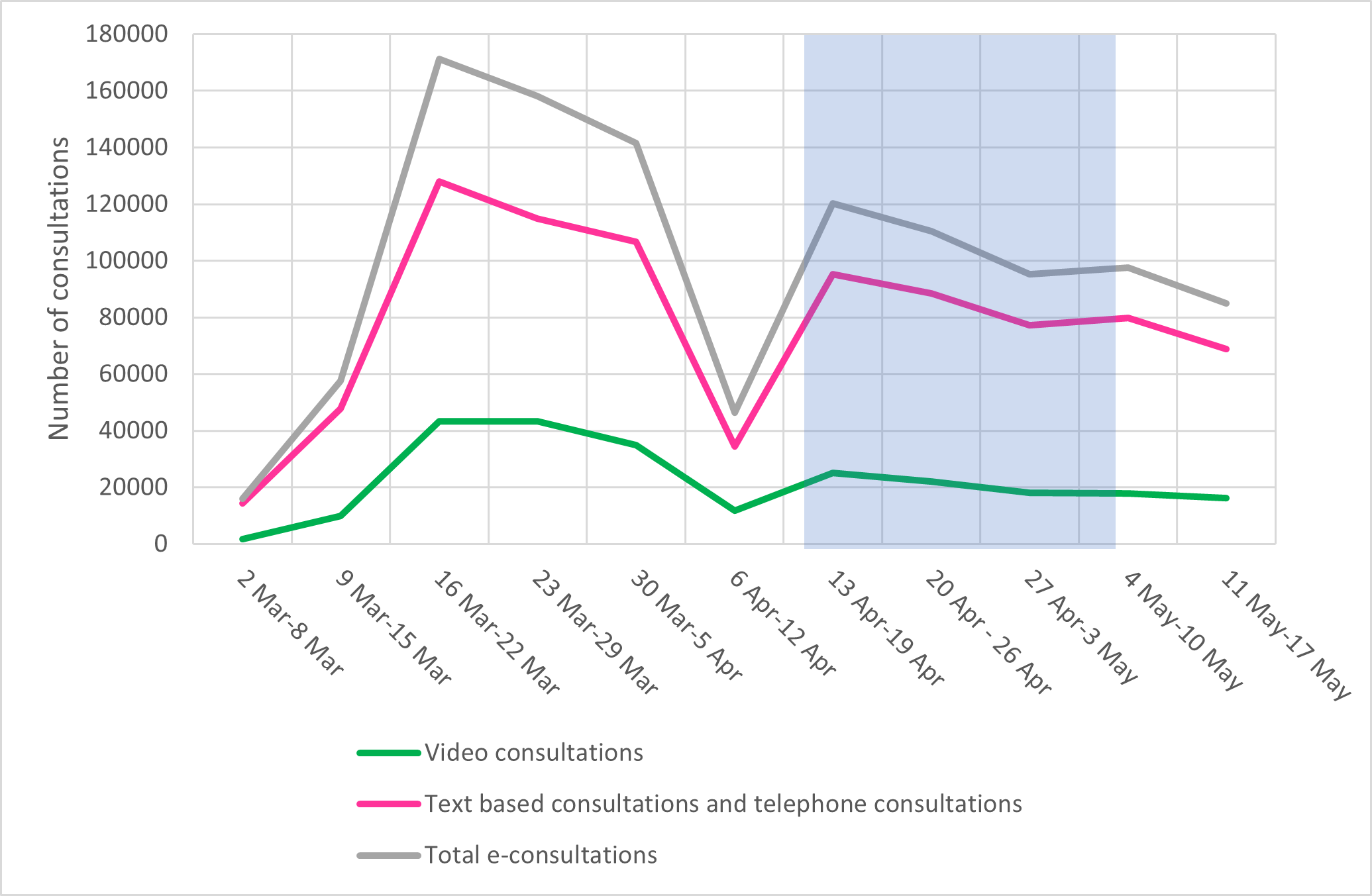

Supplement: Multimedia Appendix 2 [file jmir_v23i2e26433_app2.png]
